# Electronic Impact of High-Energy Metal Deposition on Ultrathin Oxide Semiconductors

**DOI:** 10.1021/acs.nanolett.4c05333

**Published:** 2025-01-21

**Authors:** Yi-Yu Pan, Min-Ju Kuo, Shih-Chieh Chen, Tanveer Ahmed, Robert Tseng, Chi-Chung Kei, Tsung-Te Chou, Che-Chi Shih, Wei-Yen Woon, Szuya Sandy Liao, Chi Chen, Der-Hsien Lien

**Affiliations:** † Institute of Electronics, National Yang Ming Chiao Tung University, Hsinchu 300093, Taiwan; ‡ Research Center for Applied Sciences, Academia Sinica, Taipei 115201, Taiwan; § Taiwan Instrument Research Institute, National Applied Research Laboratories, Hsinchu 300092, Taiwan; ∥ Research & Development, Taiwan Semiconductor Manufacturing Company, Hsinchu 300096, Taiwan; ⊥ Department of Electrophysics, National Chiao Tung University, Hsinchu 300093, Taiwan

**Keywords:** ultrathin oxide semiconductor, indium oxide (In_2_O_3_), high-energy metal deposition, localized annealing, threshold voltage, negative contact resistance, metal−semiconductor interface

## Abstract

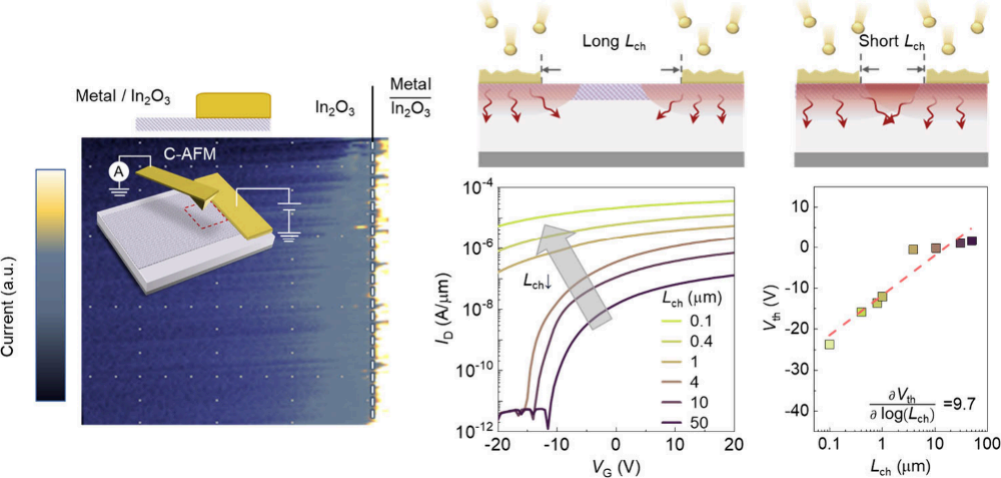

High-energy metal deposition significantly impacts the performance and reliability of two-dimensional (2D) semiconductors and nanodevices. This study investigates the localized annealing effect in atomically thin In_2_O_3_ induced during high-energy metal deposition. The localized heating effect alters the electronic performance of In_2_O_3_ devices, especially in shorter channel devices, where heat dissipation is further constrained. This effect creates a conductivity gradient along the In_2_O_3_ device with higher conductivity near the metal contact, as observed by conductive atomic force microscopy (C-AFM). This gradient leads to a pronounced threshold voltage (*V*_th_) shift as the channel length (*L*_ch_) decreases, resembling a short-channel effect but one driven by thermal mechanisms rather than conventional mechanisms. Furthermore, metals with higher latent heats can exacerbate these effects. We also show that reversing the deposition sequence and postdeposition oxygen annealing effectively suppress *V*_th_ shifts across different *L*_ch_. This work offers key insights into controlling thermal effects during fabrication to improve ultrathin oxide transistor performance.

Reducing the thickness of semiconductors is a critical strategy to enable continuous scaling in transistor technologies, as it improves electrostatic control that mitigates short-channel effects during device miniaturization.^1^ However, thinning thickness typically leads to a decrease in carrier mobility for conventional semiconductors, especially when it approaches nanoscale thicknesses.^2−4^ For instance, the mobility of silicon drops from ∼300 cm^2^ V^–1^ s^–1^ at a thickness of 6 nm to lower than 10 cm^2^ V^–1^ s^–1^ at 2 nm.^5^ To address this issue, emerging materials, such as two-dimensional (2D)^6−11^ and quasi-2D^12−14^ semiconductors, have garnered significant interest. As a promising option with ultimate thickness scaling capability, 2D transition metal dichalcogenides (TMDCs) like MoS_2_ and WSe_2_ have demonstrated high mobilities (>100 cm^2^ V^–1^ s^–1^) and excellent on/off ratios (>10^8^) at their monolayer thickness limit.^15−18^ Recently, ultrathin In_2_O_3_ has emerged as a potential candidate for the channel material of transistors due to its excellent transport properties, with recent research demonstrating high mobility (>100 cm^2^ V^–1^ s^–1^) and high on/off ratio (>10^10^) at a thickness of 2 nm.^19−23^ These materials preserve high performance at nanometer-scale thicknesses, offering enhanced electrostatic control and scalability, making them ideal candidates for next-generation electronic devices.

On the other hand, when semiconductors are made ultrathin, their physical properties become increasingly susceptible to processing conditions and environmental exposure.^24^ This increased vulnerability arises from their large surface-to-volume ratio and the lack of bulk material to protect against external factors. In the case of 2D materials, high-energy metal deposition techniques often involve atom or cluster bombardment and intense localized heating at the contact region, which can disrupt the crystal lattice at or near the interface.^25−28^ This damage at the 2D surface was previously associated with the Fermi-level pinning effect, which causes high contact resistance (*R*_c_) despite efforts in contact engineering. In addition to the damage during fabrication process, postprocess factors such as the adsorption of gases, exposure to chemicals, temperature fluctuations, and illumination can also adversely impact the electrical properties of ultrathin semiconductors.^29,30^ In the case of oxide semiconductors, due to their temperature instability, the heat generated during metal deposition can cause n-doping in oxide semiconductors, leading their threshold voltage (*V*_th_) to shift drastically, severely affecting the electrical properties.^31,32^

In this work, we investigate the electronic impact of high-energy metal deposition on ultrathin In_2_O_3_ transistors, specifically focusing on the localized annealing effects caused by metal condensation during fabrication. The study reveals that metals with a higher latent heat of condensation induce significant localized heating at the metal–semiconductor interface. Using conductive atomic force microscopy (C-AFM), a clear conductivity gradient was observed, with a higher conductivity near the metal contact due to localized annealing. These effects are more severe in shorter channel lengths (*L*_ch_), where heat accumulation leads to a pronounced negative *V*_th_ shift due to n-type doping effects. Additionally, the *V*_th_ shift across varying results in an observed negative contact resistance when the transfer length method (TLM) is used for contact resistance extraction. This also leads to an abnormal short-channel effect, with the *V*_th_ shift occurring at much longer than typically expected in conventional short-channel behavior. We also show that reversing the fabrication process by depositing the metal first, followed by forming the In_2_O_3_ layer, effectively eliminates the annealing effect and reduces the conductivity gradient. Additionally, postdeposition oxygen annealing further mitigates these effects. Our study provides critical insights into controlling thermal effects during fabrication to enhance the reliability of ultrathin oxide semiconductor transistors.

The electronic properties of In_2_O_3_ are highly sensitive to annealing, as oxygen vacancies (V_o_), acting as both donors or electron traps, are alterable during thermal treatment.^32^Figure 1a shows that when the annealing temperature is increased from 100 to 225 °C, the *V*_th_ of ultrathin In_2_O_3_ transistors (2 nm thick) with a 30 nm back-gate SiO_2_ dielectric (Figure 1b) shifts significantly from +5 to −26 V. This *V*_th_ shift corresponds to a substantial increase in the conductivity of In_2_O_3_, rising from 187 to 6870 S m^–1^. The annealing effect also occurs during the fabrication process when electrodes are deposited on the In_2_O_3_. As the electrode formation usually involves high-energy metal deposition, high latent heat of the evaporated metal could cause heat accumulation at the deposited regions of the semiconductor, leading to an increase in local temperature.^33^ This phenomenon has also been observed in previous studies on III–V compound semiconductors, where high-energy atom bombardment resulted in local temperature increases, inducing recrystallization and generating defects such as vacancies and interstitials.^34^ Such heat accumulation and elevated temperatures are particularly pronounced in a transistor with an ultrathin body (UTB), as the surrounded dielectric layers have relatively low thermal conductivity^35^ (<10 W m^–1^ K^–1^ for dielectric layer compared to >100 W m^–1^ K^–1^ for metal) that impedes heat dissipation and causes rapid temperature increases.^36^ Furthermore, the increased heat accumulation with reduced channel thickness is primarily attributed to the decrease in thermal conductivity as the thickness decreases, leading to elevated temperatures, as illustrated in Figure S1. Elevated temperatures can significantly modulate the semiconductor properties, affecting the performance of devices. For example, UTB structures like silicon on insulator (SOI) exhibit self-heating effects, which result in reduced carrier mobility due to enhanced phonon scattering.^37−39^

**Figure 1:**
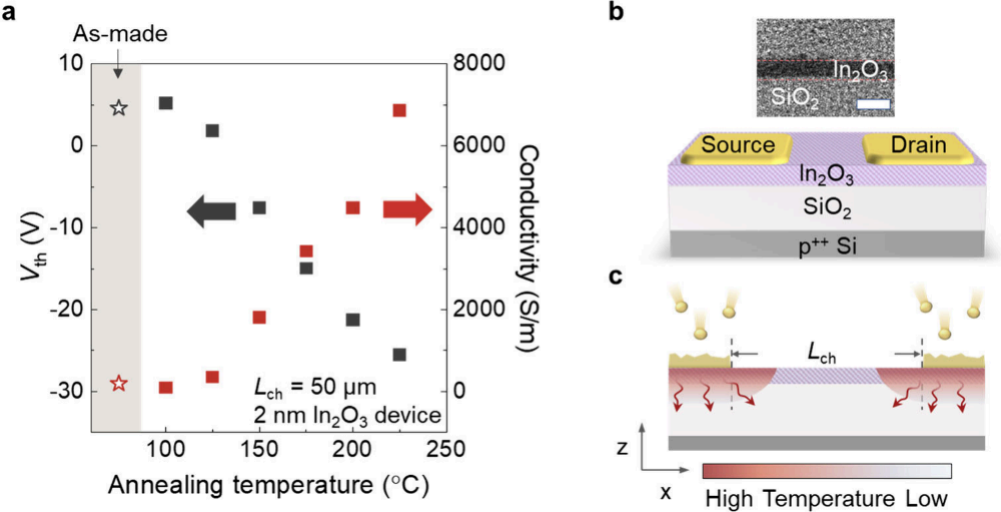
Electronic impact of high-energy deposition on In_2_O_3_ transistors. (a) The negative *V*_th_ shifts and increased conductivities after annealing in N_2_ at different temperatures for 30 min, with *L*_ch_ of 50 μm. The extraction of conductivity is performed at *V*_G_ = 0 V and *V*_D_ = 0.1 V. (b) Schematic of the In_2_O_3_ transistor. Inset, TEM image of 2 nm In_2_O_3_. (c) Illustrations of the elevated temperature in devices with long *L*_ch_ during the thermal evaporation process.

The temperature distributions during metal deposition for devices with varying *L*_ch_ values were obtained through simulations, as illustrated in Figure S2. The elevated temperature is highest at the deposited contact region due to restricted heat dissipation in the UTB structure, and the temperature gradually decreases along the channel, as shown in Figure 1c. Consequently, the transistor experiences varying levels of annealing along the channel, leading to a nonuniform annealing effect. Due to the intensive heating near the metal contact, the localized annealing effect causes an increase in conductivity of In_2_O_3_ near the deposited region, effectively resulting in a permanent n-type doped-like behavior. Note that two mechanisms contribute to the observed conductivity change in In_2_O_3_. For relatively low temperature annealing (<300 °C), it could trigger the charge transfer process between the adsorbed oxygen and the In_2_O_3_, resulting in reversible changes to its conductivity.^40^ In contrast, higher temperature annealing (>300 °C) caused by deposited metal, as indicated by simulations in Figure S2, induces irreversible lattice relaxation, altering V_o_ charge states, as discussed in several studies.^41,42^ V_o_ exists in V_o_^0^, V_o_^+1^, and V_o_^+2^ states, with only V_o_^+2^ acting as a shallow donor to increase carrier concentration. In as-made In_2_O_3_, V_o_^0^ and V_o_^+1^ dominate. High temperature annealing induces lattice relaxation, converting the V_o_ charge state to V_o_^+2^, thereby permanently increasing the carrier concentration and conductivity.

The reduced annealing effect along the channel creates a conductivity gradient, which can be directly observed in the C-AFM measurements shown in Figure 2. Here, C-AFM was utilized to map the spatial conductivity variation of the In_2_O_3_, with a focus on the semiconductor–metal contact region. Figure 2a shows that C-AFM is used to measure the current flowing from a metal electrode to a probe positioned on the channel, with the probe scanning the entire channel to determine the current magnitude at each location. Figure 2b presents the simulation of current flow, accounting for the current-spreading effect, and illustrates the current-spreading model used for conductance extraction (details in the Supporting Information). Figure 2c,e shows that the measured current decreases as the probe moves away from the contact. The bottom panel of Figure 2e shows that the conductivity at the metal–semiconductor junction is 0.6 S m^–1^, gradually decreasing with distance from the contact until it reaches a value of 0.006 S m^–1^. The high-conductivity region extends approximately 400 nm from the contact, consistent with the heat dissipation length shown in the simulation, as shown in Figure S3. The temperature difference between the center of the channel and the metal–semiconductor junction is ∼200 °C, resulting in an increased conductivity near the metal–semiconductor junction that is about 2 orders of magnitude higher than the conductivity of the center region.

**Figure 2:**
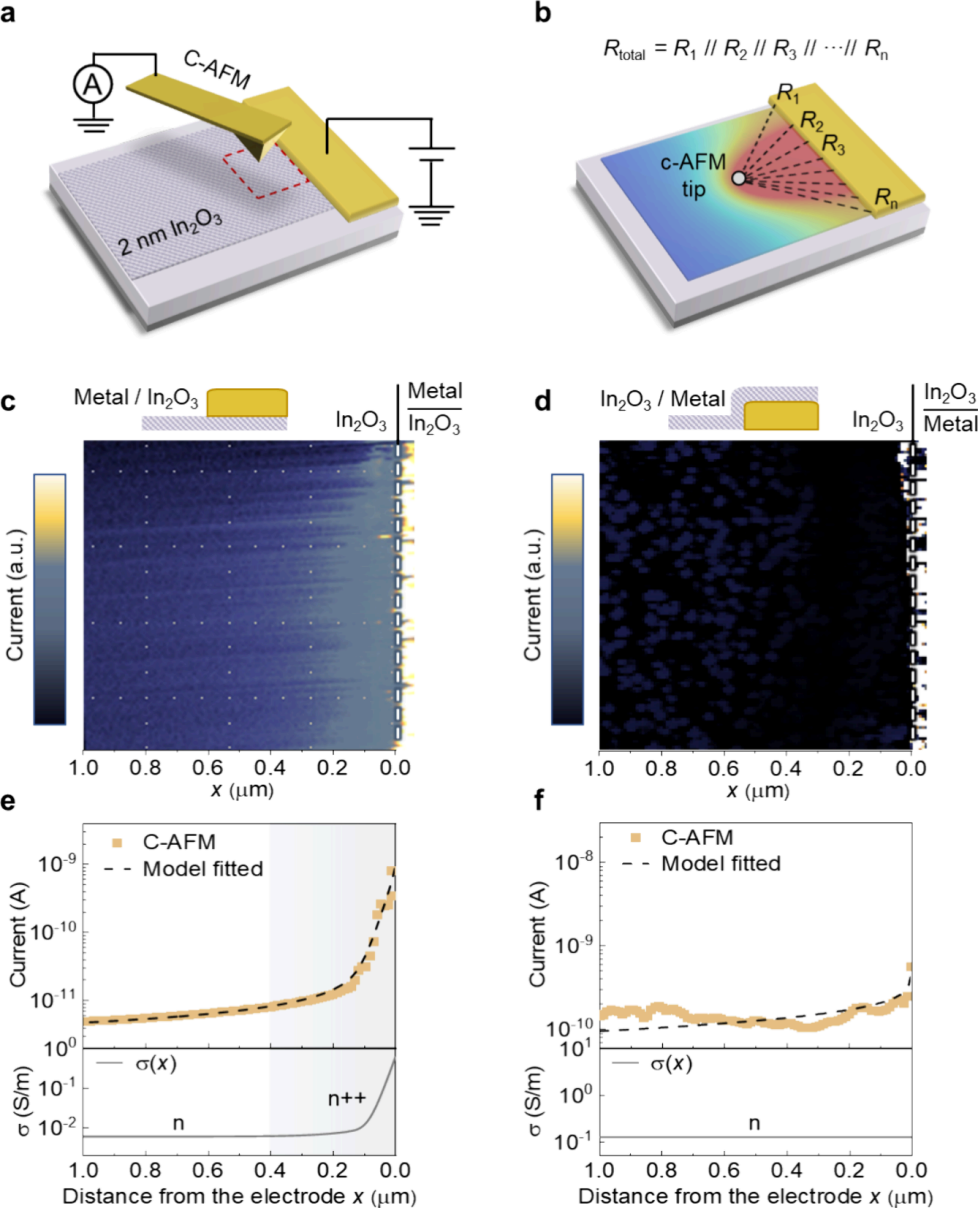
Localized annealing effects observed in devices with an *L*_ch_ of 5 μm using C-AFM. (a) Schematic of C-AFM experimental setup, with a constant voltage applied to the metal electrode and the grounded probe scanning across the channel. (b) Simulation of current flow and equivalent total resistance across the channel based on the current-spreading model. (c) C-AFM current measurement for the device with the deposition of In_2_O_3_ followed by metal deposition. Here, the source electrode is grounded. (d) C-AFM current measurement for the device with the deposition of metal followed by In_2_O_3_. (e) The measured current as a function of position *I*(*x*) measured by C-AFM and calculated conductivity distribution from (c). (f) The measured current as a function of position *I*(*x*) measured by C-AFM and calculated conductivity distribution from (d).

To verify the origin of the conductivity gradient, we reversed the fabrication process by first depositing the metal on the dielectric, followed by forming an In_2_O_3_ layer on top of the metal electrodes. As In_2_O_3_ was deposited last, it effectively prevented the thermal effects caused by the deposited metal. The C-AFM measurement (Figure 2d,f) reveals that the conductivity of the In_2_O_3_ channel is nearly uniform throughout the channel. The extracted conductivity, as shown in the bottom panel of Figure 2f, retains a constant value of ∼0.1 S m^–1^ along the whole channel, indicating the absence of localized annealing effects since In_2_O_3_ was deposited last.

We then investigated the impacts of localized annealing on the performance of the In_2_O_3_ transistors. The lateral conductivity gradient along the In_2_O_3_ channels, induced by localized annealing, can significantly impact the transistor performance, particularly the measured *V*_th_. In devices with two electrode contacts closely formed, i.e., with shorter *L*_ch_, the localized annealing effect is amplified as the heat generated by adjacent contacts accumulates, leading to higher elevated temperatures, as illustrated in Figure 3a (simulated results are shown in Figure S2). This amplified annealing effect in shorter *L*_ch_ devices leads to a stronger n-type doping effect, resulting in higher conductivity. Figure 3b shows the current across the entire channel of devices with *L*_ch_ values of 0.1 and 0.8 μm measured by C-AFM. The shorter channel devices exhibit conductivity approximately 1–2 orders of magnitude higher than that of the longer channel devices, consistent with the transfer characteristics (*I*_D_–*V*_G_) data for the respective *L*_ch_. The transfer characteristics reveal a negative *V*_th_ shift from 1.7 to −23.7 V as the *L*_ch_ is reduced from 50 μm to 100 nm, as shown in Figure 3c and Figure S4 (see the Experimental Section for the *V*_th_ extraction). Note that the “measured” *V*_th_ of the device reflects a macroscopic effect, revealing the overall characteristics of the nonuniform semiconductor channel. The channel conductance is dominated by the least conductive part of the channel, which determines the “measured” *V*_th_ value of the device. In comparison between long- and short-channel devices, the less conductive portion in the center of the short channel, influenced by temperature-induced *E*_f_ elevation, results in a lower *V*_th_. The negative shift of *V*_th_ with reduced *L*_ch_ caused by the above-mentioned mechanism resembles the short-channel effect, which exhibits the same *V*_th_–*L*_ch_ dependence due to the overlap of the inversion region in the length scaled transistors. However, for 2 nm thick In_2_O_3_, the short-channel effect should occur when *L*_ch_ < 12 nm,^10,43^ far shorter than 1 μm when the *V*_th_ shift is observed in In_2_O_3_ devices (Figure S5). Furthermore, the drain-induced barrier lowering (DIBL) effect is not observed in In_2_O_3_ transistors with shortest *L*_ch_ values of 100 nm in this work, indicating the observed *V*_th_ shift is not caused by the conventional short-channel effects (Figure S6).

**Figure 3:**
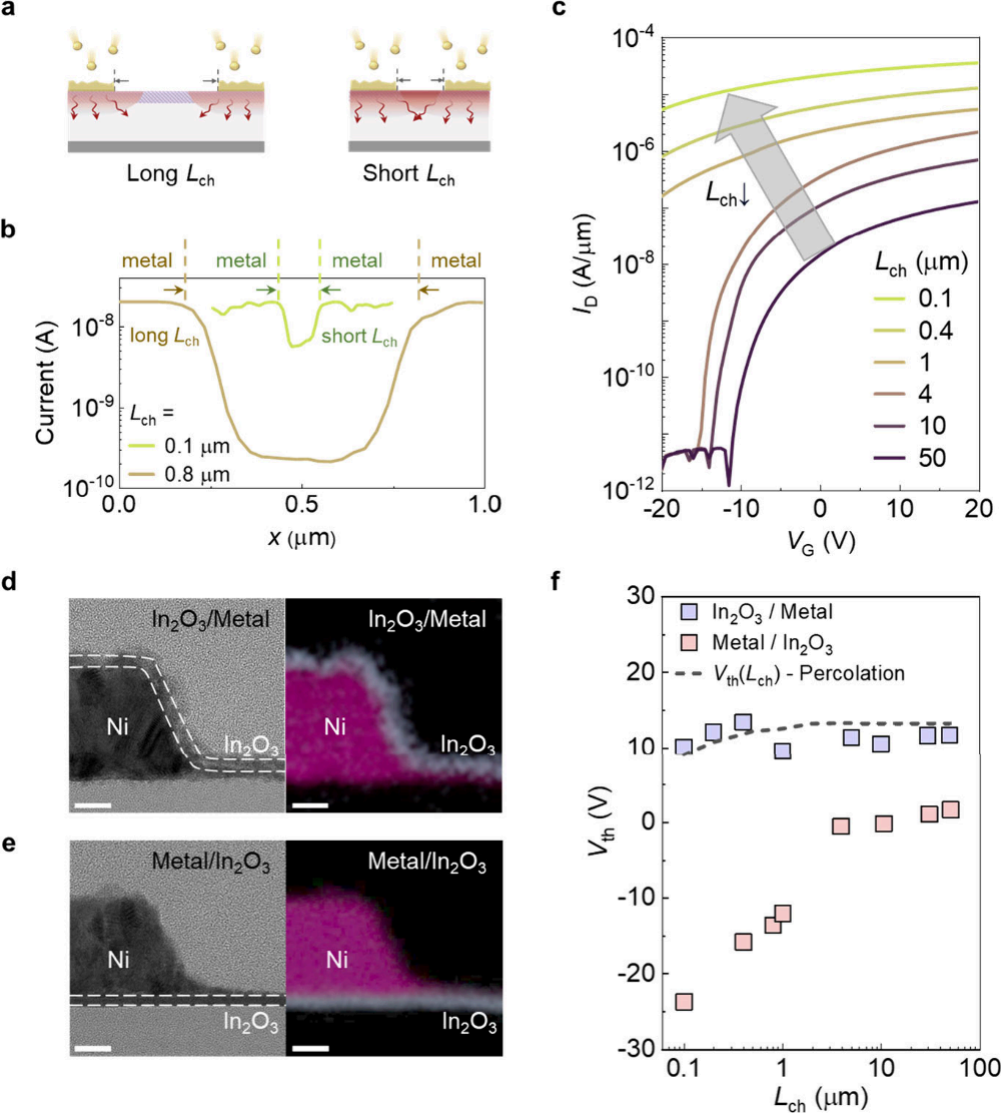
Influence of localized annealing effects on the transistor and verification of metal deposition-induced localized annealing. (a) Illustrations of the elevated temperature in devices with long and short *L*_ch_ during the thermal evaporation process. (b) The measured current as a function of position *I*(*x*) across the entire channel measured by C-AFM for devices with *L*_ch_ of 0.1 and 0.8 μm. Here, both sides of the electrode are grounded. (c) Transfer characteristics of ultrathin In_2_O_3_ transistors with different *L*_ch_ under *V*_D_ = 0.1 V. (d) TEM image of ALD-deposited In_2_O_3_ after metal deposition, with false-color EDS elemental mapping of Ni and In_2_O_3_. (e) TEM image of metal deposition process after ALD-In_2_O_3_, with false-color EDS elemental mapping of Ni and In_2_O_3_. (f) The *V*_th_ of devices with reversed deposition sequences of metal and In_2_O_3_.

To compare results unaffected by localized annealing, we prepared a control In_2_O_3_ device by reversing the deposition process sequence. The different deposition orders of In_2_O_3_ and electrodes are clearly visible in the TEM images shown in Figure 3d,e. The results in Figure 3f show that the *V*_th_ of the control devices, where the metal was deposited first, followed by In_2_O_3_, remained unchanged across varying *L*_ch_. Note that the negative *V*_th_ shift with reduced *L*_ch_ follows the same trend as the percolation threshold shift with reduced *L*_ch_, but the observed *V*_th_ shift is predominantly caused by the deposition-induced annealing effect when the metal is deposited on In_2_O_3_.

The increases in channel conductivity with decreased *L*_ch_ can be clearly observed by using TLM, as shown in Figure 4a. In a device with an *L*_ch_ of 50 μm, the sheet resistance (*R*_sh_) is 663 kΩ □^–1^, while in a device with an *L*_ch_ of 100 nm, the *R*_sh_ decreases significantly to 18 kΩ □^–1^. This result indicates that *R*_sh_ decreases with reduced *L*_ch_ due to the enhanced annealing effects, consistent with the *V*_th_ shift trend shown in the transfer characteristics. Interestingly, because TLM assumes constant *R*_sh_ for all *L*_ch_, the *L*_ch_-dependent *R*_sh_ introduces errors in extracting *R*_c_, leading to a significant underestimation of its values. As the conductivity increases with shorter *L*_ch_, regular extrapolation yields a negative *R*_c_ of −3 kΩ μm, as shown in the inset of Figure 4a (see the Experimental Section). Note that the ultralow *R*_c_ of In_2_O_3_ devices was observed in other studies and was previously attributed to negative Schottky barriers.^44^ To adjust the TLM measurement for correct *R*_c_ extraction, a fixed drive voltage (*V*_G_ – *V*_th_) is applied to compensate for the varying *R*_sh_ with different *L*_ch_. As *R*_c_ varies with carrier concentration determined by the drive voltage, fixing the drive voltage at 5, 10, and 15 V results in *R*_c_ values of 7.8, 5.1, and 4.2 kΩ μm, respectively, as shown in Figure 4b. These low *R*_c_ values reflect the annealing effect, which heavily dopes the contact region and explains why In_2_O_3_-based devices can approach the quantum limit of *R*_c_. Note that although fixing the drive voltage for different *L*_ch_ values to a certain degree improves the accuracy of the *R*_c_ extraction, it is still inaccurate for cases with localized annealing effects. This is because regardless of the drive voltage, the nonuniform conductivity remains in the channel, which violates the assumptions of the TLM model and leads to an inaccurate *R*_c_.

**Figure 4:**
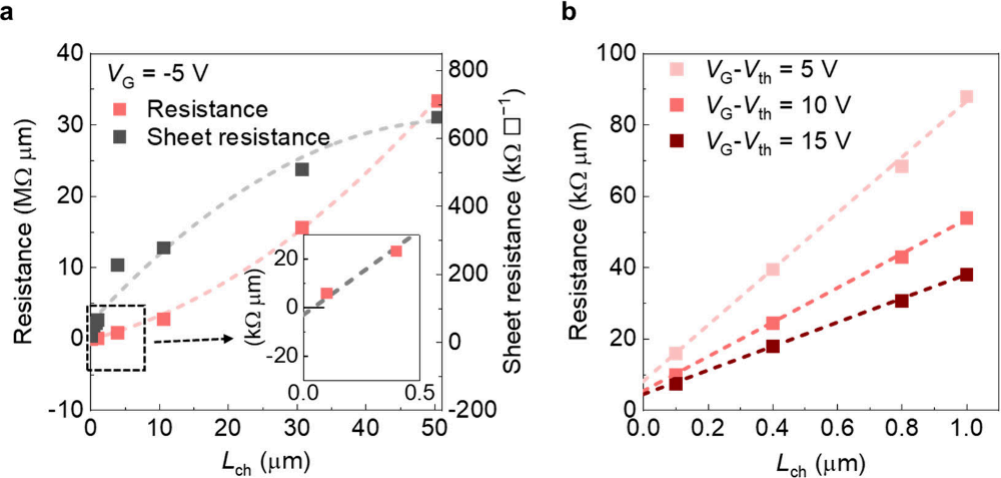
Influence of localized annealing effects on *R*_c_ and *R*_sh_ extracted using TLM. (a) Resistance and *R*_sh_ extracted from Figure 3b using TLM. Inset, *R*_c_ is −3 kΩ μm. (b) *R*_c_ extraction of 7.8, 5.1, and 4.2 kΩ μm by TLM at the *V*_G_ – *V*_th_ of 5, 10, and 15 V, respectively.

The intensity of the localized annealing effect is determined by the latent heat of the deposited metals. During deposition, as the evaporated metal condenses, the resulting temperature increase is governed by its latent heat. As such, we deposited three metals with different latent heats of condensation, namely, Bi (190.3 kJ/mol), Ni (396.5 kJ/mol), and Mo (635.5 kJ/mol), on In_2_O_3_, as shown in Figure 5a–c. Here, we define ∂*V*_th_/∂log(*L*_ch_) to quantify the severity of the localized annealing effect by measuring the magnitude of the *V*_th_ shift across different *L*_ch_. As the value approaches zero, *V*_th_ becomes increasingly independent of *L*_ch_. Conversely, an increase in this value will result in a greater influence of *L*_ch_ on *V*_th_. The values of ∂*V*_th_/∂log(*L*_ch_) for Bi, Ni, and Mo are 7.1, 9.7, and 16.7, respectively, consistent with the order of their latent heats of condensation, as shown in Figure 5d. When depositing metals with a lower heat of condensation, the resulting local heat accumulation is lower, leading to a smaller annealing effect and a smaller *V*_th_ shift. Conversely, depositing metals with a higher heat of condensation results in a stronger annealing effect, leading to a larger *V*_th_ shift. This trend indicates that the impact of the localized heating effect is directly related to the latent heats of the evaporated metals, which determine the elevated temperatures.

**Figure 5:**
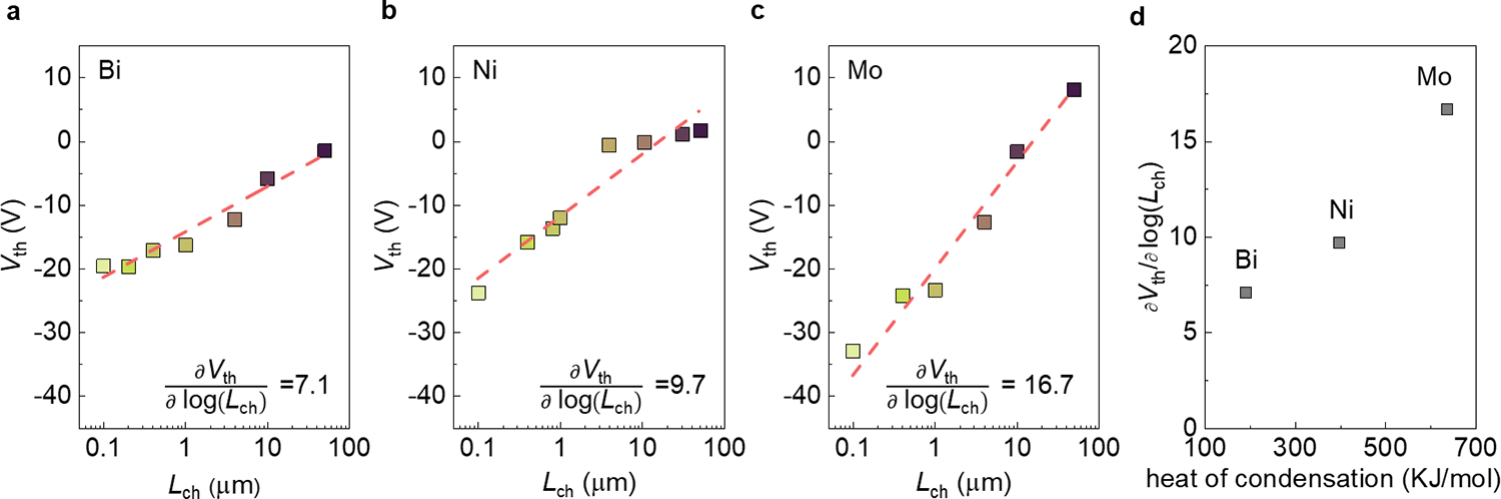
Comparison of the localized annealing effects in devices with different metal electrodes. (a) The *V*_th_ of devices with Bi electrodes with different *L*_ch_. (b) The *V*_th_ of devices with Ni electrodes with different *L*_ch_. (c) The *V*_th_ of devices with Mo electrodes with different *L*_ch_. (d) Comparison of *∂*V*_th_/∂log(*L*_ch_)* with the heat of condensation of Bi, Ni, and Mo.

In addition to the latent heat of metals, the localized annealing effects are also influenced by the operational parameters and conditions of the deposition process. We employed the sputter deposition method to form the Ni contact, as shown in Figure S7. Due to the reduced deposition temperature during sputtering,^45^ the value of ∂*V*_th_/∂log(*L*_ch_) for the same metal decreases from 9.7 to 7.3 when comparing thermal evaporation to sputtering. Additionally, the deposition rate also affects the magnitude of *V*_th_ shifts, with localized annealing effects becoming more pronounced at higher deposition rates, as shown in Figure S8.

We also show that postdeposition oxygen annealing can effectively mitigate the conductivity gradient in transistors, as shown in Figure S9. When the device is annealed in an oxygen-rich environment, oxygen can be adsorbed onto the surface of the channel or diffused into the channel to fill V_o_, thereby allowing the *V*_th_ to be redefined for devices with different *L*_ch_ values. After being annealed in oxygen at 200 °C for 30 min, ∂*V*_th_/∂log(*L*_ch_) changes from 10.4 to 2.6, and the *V*_th_ of the device with an *L*_ch_ of 100 nm becomes −5.8 V, decreasing by 14 V. These results demonstrate that proper treatments and design modifications of ultrathin oxide semiconductor devices can significantly mitigate the effects of localized heating, enhancing electronic performance, reliability, and noise margins in integrated circuits.

This study demonstrated that localized heating during high-energy metal deposition induces a lateral n-doping gradient in ultrathin In_2_O_3_ semiconductor channels, causing an *L*_ch_-dependent *V*_th_ shift. Shorter channels exhibit more pronounced negative shifts due to enhanced localized annealing effects, as confirmed by C-AFM. Mitigation strategies such as reversing the deposition sequence, utilizing metals with lower latent heats of condensation, or applying postdeposition oxygen annealing effectively reduce these thermal effects and lead to more consistent *V*_th_. Understanding and controlling these fabrication-induced thermal effects are crucial for designing ultrathin oxide semiconductor transistors with improved performance and reliability. This work provides valuable insights into enhancing device consistency and reducing variability in next-generation electronic devices.

## Experimental Section

### Device Fabrication

The device fabrication started with a heavily phosphorus-doped Si wafer covered with 30 nm SiO_2_ and 2 nm In_2_O_3_ thin film formed by ALD. The active areas of transistors were defined by photolithography (for *L*_ch_ > 1 μm) and e-beam lithography (for *L*_ch_ < 1 μm) and etched with dilute HCl (HCl:H_2_O = 1:50) for 10 s. The metal electrodes were formed by electron-beam evaporation of Ni.

### Annealing Processes

The fabricated device was placed in a custom chamber, ventilated with nitrogen or oxygen, and heated on a hot plate. The gas flow was approximately 1 L/min, and the pressure was around 1 atm.

### Extraction of *R*_c_ and *R*_sh_ through TLM

In the case of thin-film transistors, the total resistance (*R*_tot_, in units of kΩ μm) of the transistor is calculated based on the drain voltage and drain current. The *R*_tot_ of the transistor is primarily composed of *R*_c_ (in units of kΩ μm) of the electrode–channel interface and the resistance of the channel itself, which is determined by *R*_sh_ (in units of kΩ per square, kΩ □^–1^) and *L*_ch_. This can be expressed as *R*_tot_ = *R*_c_ + *R*_sh_*L*_ch_. When *R*_c_ and *R*_sh_ are independent of *L*_ch_, *R*_tot_ can be plotted as a function of *L*_ch_. By performing a linear fit on the data points, it is possible to obtain a fitting line whose slope represents the *R*_sh_. Furthermore, by extrapolating this line to the *x*-axis (*L*_ch_ = 0), we can determine the *R*_c_.

### Process for Different Metal Deposition

In this study, we utilized an evaporator and a sputtering machine to deposit metal, evacuating the chamber to a pressure of 4 × 10^–6^ Torr prior to deposition with both machines depositing 30 nm of material as the electrodes. The deposition of Ni was achieved by the evaporator and the sputtering machine at a deposition rate of 0.1 Å/s. The comparative results are presented in Figure S7. The outcomes of Ni deposition by the evaporator with deposition rates of 0.1 and 1 Å/s are also displayed in Figure S8. Bi and Mo were deposited by the evaporator, with a deposition rate of 0.1 Å/s maintained.

### TEM

The samples were prepared with devices of In_2_O_3_/Ni and Ni/In_2_O_3_ by a Helios 5 DB-FIB instrument. Then TEM was performed using a JEOL 2800F instrument under 200 keV.

### Device Characterization

Electrical characterization was carried out using an Agilent B2902B source and a probe station at room temperature. The *V*_th_ of ultrathin In_2_O_3_ transistors was extracted by the linear extrapolation method. For MOSFET in the linear regime (*V*_D_ < *V*_G_ – *V*_th_), we used the equation *I*_D_ = μ*C*_ox_(*V*_G_ – *V*_th_ – )*V*_D_, where μ is the mobility of a transistor and *C*_ox_ is the oxide capacitance. We extrapolated the *I*_D_–*V*_G_ curve to the point where *I*_D_ equals zero and added *V*_D_/2 to obtain *V*_th_.

### Conductive Atomic Force Microscopy

The C-AFM experiments were performed by using a custom-built AFM equipped with a Nanonis controller. During the experiments, the nanoelectronic properties of the samples were measured in contact mode at room temperature. The signal was amplified by a low-noise current preamplifier (DLPCA-200, Femto), and platinum-deposited cantilevers (OMCL-AC240TM, Olympus) were used for all C-AFM measurements.

### Latent Heat of Metal

During metal deposition via PVD, metal vapor is deposited onto the wafer surface. This involves phase transitions, where the metal changes from solid (or occasionally liquid) to vapor during evaporation and then reverts to solid as it condenses onto the wafer. Latent heat is released during phase transitions, including the heats of both vaporization and fusion, as metals undergo deposition.

## Supporting Information Available

Additional experimental data, material characterization, and simulation experiment details (conditions and parameters used in simulations, methods for calculating channel resistance in C-AFM, and related findings) (PDF)
