# Supplementary material for: Electronic Impact of High-Energy Metal Deposition on Ultrathin Oxide Semiconductors
Source: Nano Lett. 2025 Jan 21;25(7):2655–61. doi: 10.1021/acs.nanolett.4c05333 (PMC11849035; doi:10.1021/acs.nanolett.4c05333)
Supplement: Supplementary file 1 — nl4c05333_si_001.pdf [file nl4c05333_si_001.pdf]

Supporting Information

Electronic Impact of High-Energy Metal Deposition on Ultrathin Oxide Semiconductors

Yi-Yu Pan[1], *Min-Ju Kuo*[1,2], *Shih-Chieh Chen*[1], *Tanveer Ahmed*[1], *Robert Tseng*[1], *Chi-Chung Kei*[3], *Tsung-Te Chou*[3], *Che-Chi Shih*[4], *Wei-Yen Woon*[4], *Szuya Sandy Liao*[4], *Chi Chen*[2], *Der-Hsien Lien*[1,5*]

[1]Institute of Electronics, National Yang Ming Chiao Tung University, Hsinchu, Taiwan

[2]Research Center for Applied Sciences, Academia Sinica, Taipei, Taiwan

[3]Taiwan Instrument Research Institute, National Applied Research Laboratories, Hsinchu, Taiwan

[4]Research & Development, Taiwan Semiconductor Manufacturing Company, Hsinchu, Taiwan

[5]Department of Electrophysics, National Chiao Tung University, Hsinchu, Taiwan.

*Email: dhlien@nycu.edu.tw

Supplementary Text

1. <u>Simulation of temperature distribution along the transistor channel using COMSOL</u>

The simulation of temperature distribution is presented by a 2D model consisting of a p++ Si substrate, 30 nm SiO_2, 2 nm In_2O_3, and 20 nm Ni stack. Thermal conductivity, heat capacity, and density of materials are applied in the simulation, as shown in **Table S1**.

2. <u>Calculation of effective resistance of channel in C-AFM</u>

In C-AFM, the current flows from the metal electrode to the probe and is primarily concentrated in the triangular region formed by the tip and the electrode.[1, 2] This triangular area can be thought of as a resistor composed of several resistors connected in both series and parallel. We have simplified it to resistors connected in parallel from the probe to the electrode, each of which consists of resistors with possibly different conductivities connected in series. The resistance of this region can be calculated using the known values of the current and the electrode potential. Furthermore, by varying the position of the tip on different channels, the resistance and conductivity of each position can be determined by the following method. we can calculate conductivity, with known resistance and dimensions, because resistance is determined by the formula of

$$R = \rho \cdot \frac{L}{W\,T}$$

where ρ is the resistivity (the reciprocal of conductivity), L is the length of the resistor, defined as the distance from the tip to electrode in this case, W is the width of the resistor, T is thickness of the resistor, corresponding to the channel thickness.

	Thermal conductivity (W/m·K)	heat capacity (J/kg·K)	Density (kg/m^3)
p++ Si	149	714	2329
SiO_2	3	730	2200
In_2O_3	0.3	357	7180
Bi	7.97	122	9780
Ni	91	450	8900
Mo	138	250	10200

Table S1. Parameters used in simulation of temperature distribution.

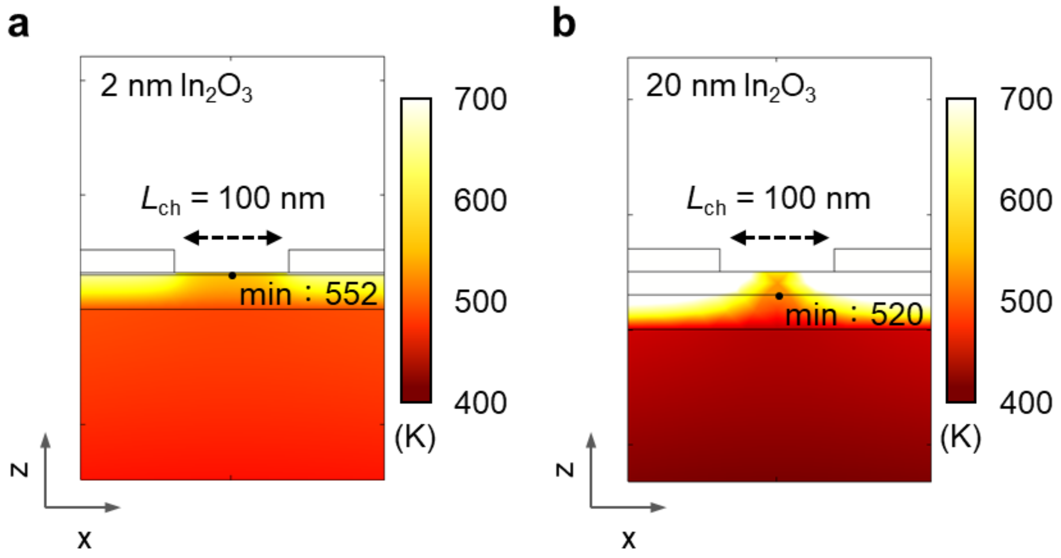

Figure S1. Comparison of simulated elevated temperatures in devices with channel thickness of 2 nm and 20 nm using COMSOL. Devices with a channel thickness of 20 nm exhibit lower temperatures at the center of the channel, demonstrating better heat dissipation efficiency.

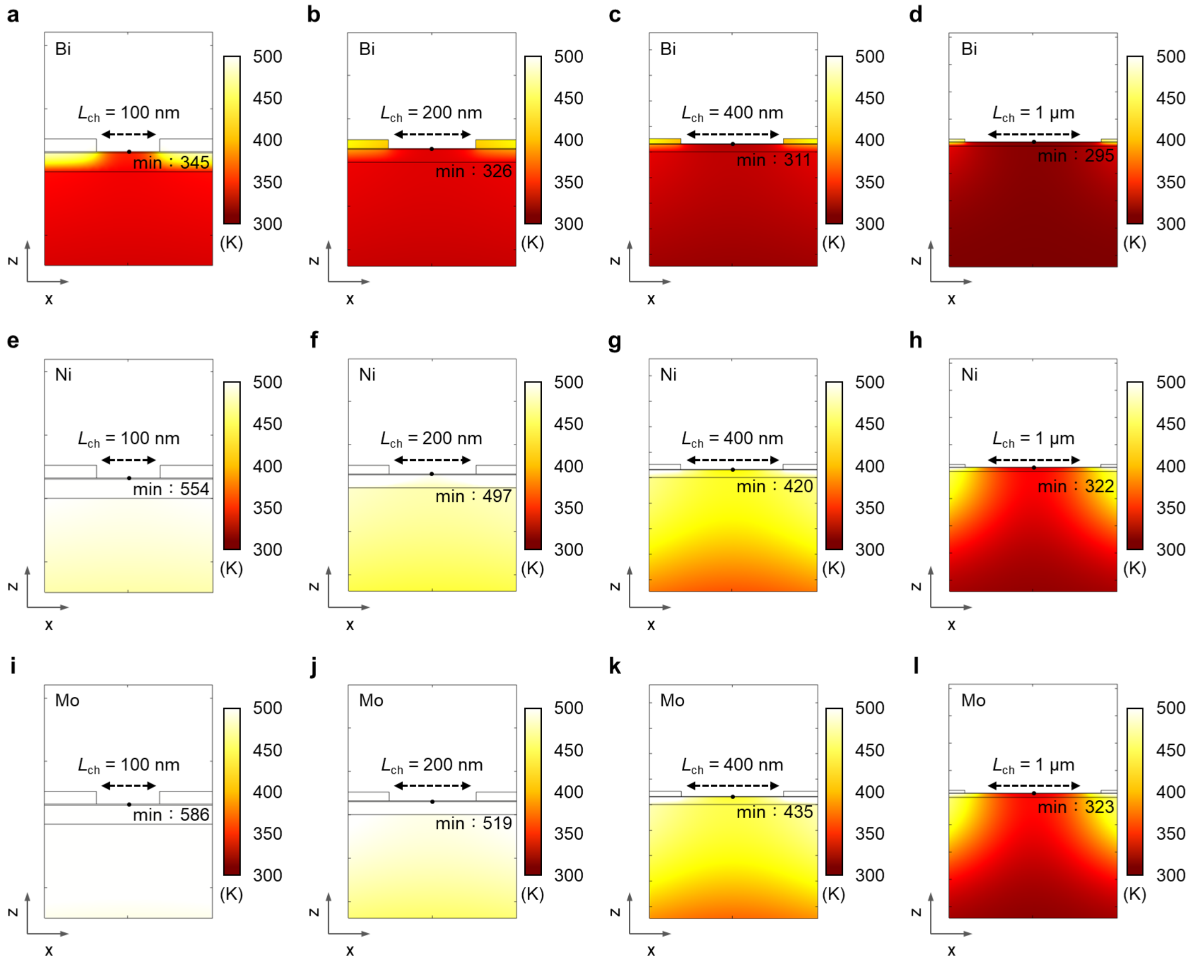

Figure S2. Simulated elevated temperatures in devices with different metals as electrodes and L_{ch} using COMSOL. (a-d) Devices with Bi electrodes. (e-h) Devices with Ni electrodes. (i-l) Devices with Mo electrodes. For devices with the same metal electrodes, longer channel lengths result in better heat dissipation efficiency and lower central temperatures.

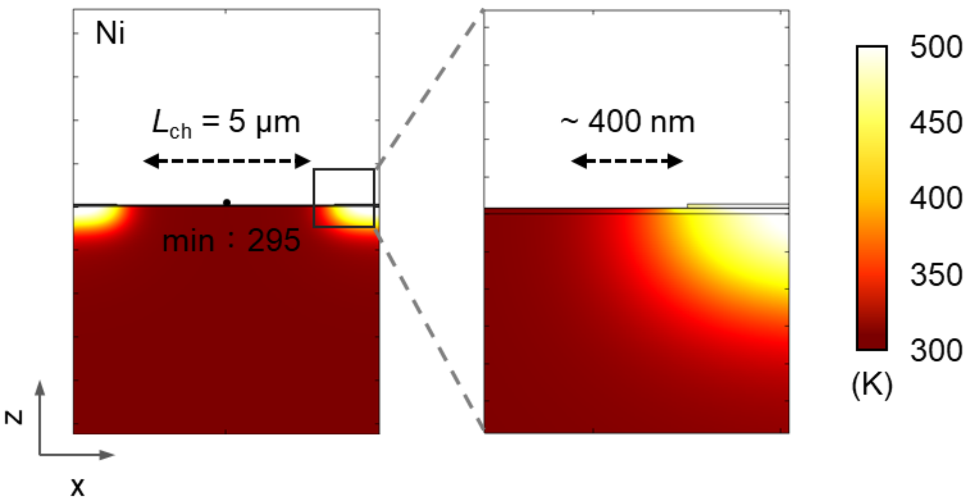

Figure S3. Simulated elevated temperatures in devices with an L_{ch} of 5 μm using COMSOL. The temperature diffusion range is approximately 400 nm, corresponding to the region of increased electron concentration shown in **Figure 2e**.

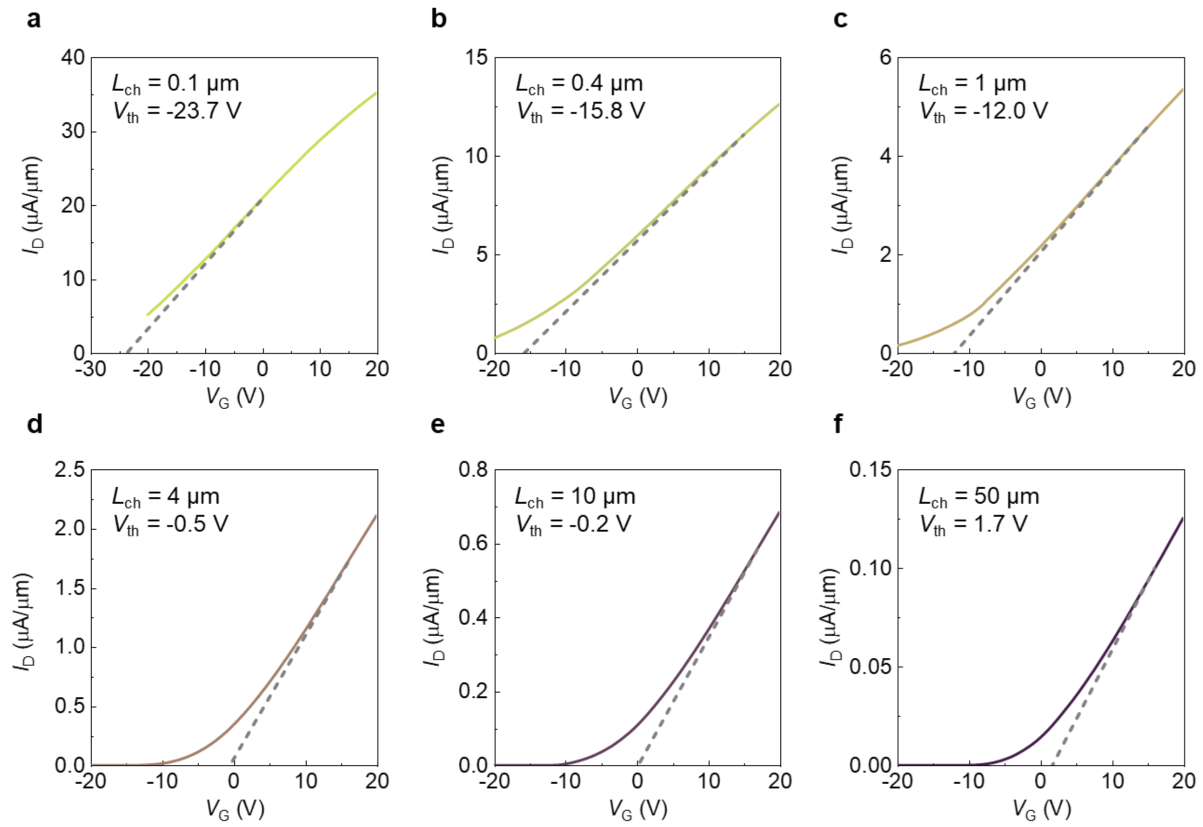

Figure S4. Transfer characteristics of ultrathin In_2O_3 transistors with different L_{ch} under V_D = 0.1 V, including extrapolated lines used for calculating the V_{th}.

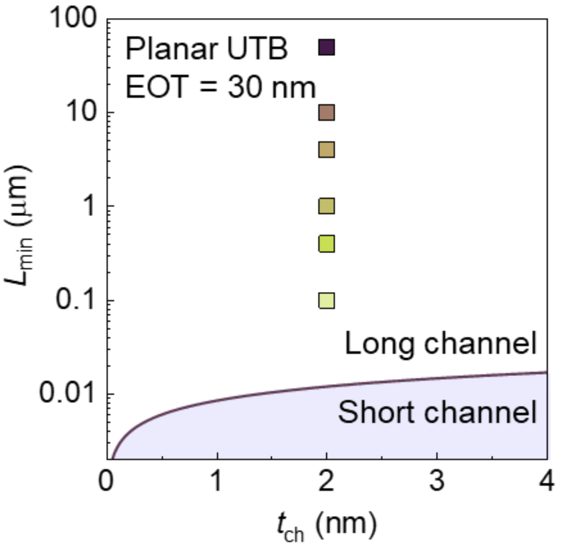

Figure S5. Calculated natural scaling length for planar ultrathin body (UTB) transistors with an equivalent oxide thickness (EOT) of 30 nm, ensuring that the transistors in this work are not affected by short-channel effects (SCEs).

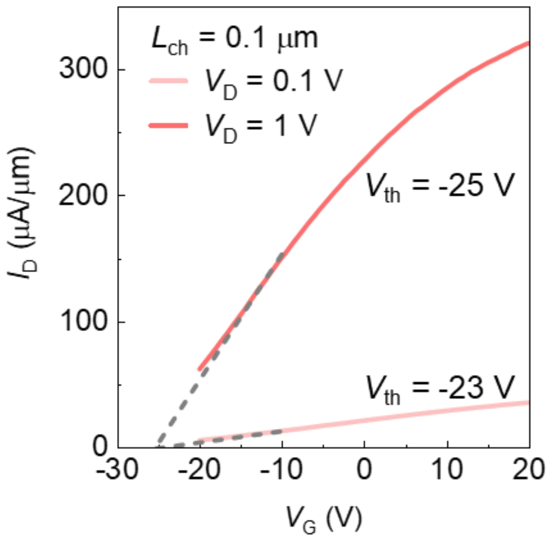

Figure S6. Transfer characteristics of an In_2O_3 device with an L_{ch} of 0.1 μm under V_D values of 0.1 V and 1 V.

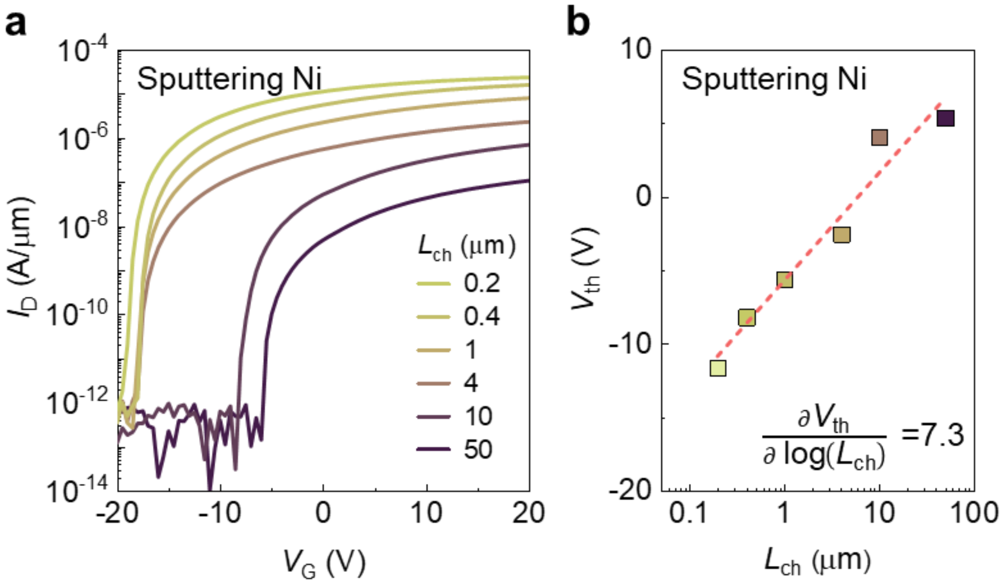

Figure S7. Influence of localized annealing effects on In_2O_3 devices with Ni electrodes deposited by sputtering. **(**a) Transfer characteristics of In_2O_3 devices with different L_{ch} under V_D = 0.1 V. (b) The V_{th} of devices with different L_{ch} under V_D = 0.1 V.

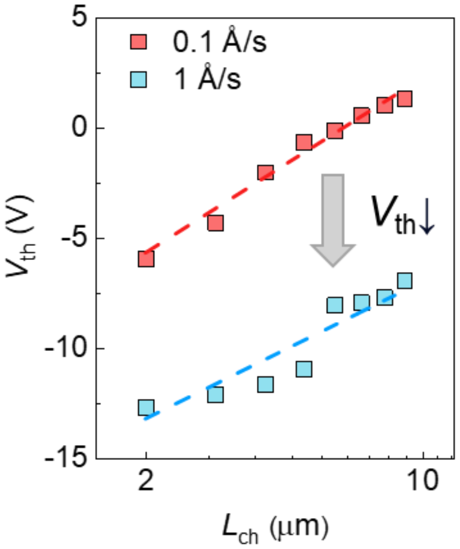

Figure S8. The V_{th} of devices at different deposition rates during the metal deposition process. The values of $\partial V_{th}/\partial \log(L_{ch})$ of devices with deposition rates of 0.1 Å/s and 1 Å/s are 11.3 and 8.9, respectively.

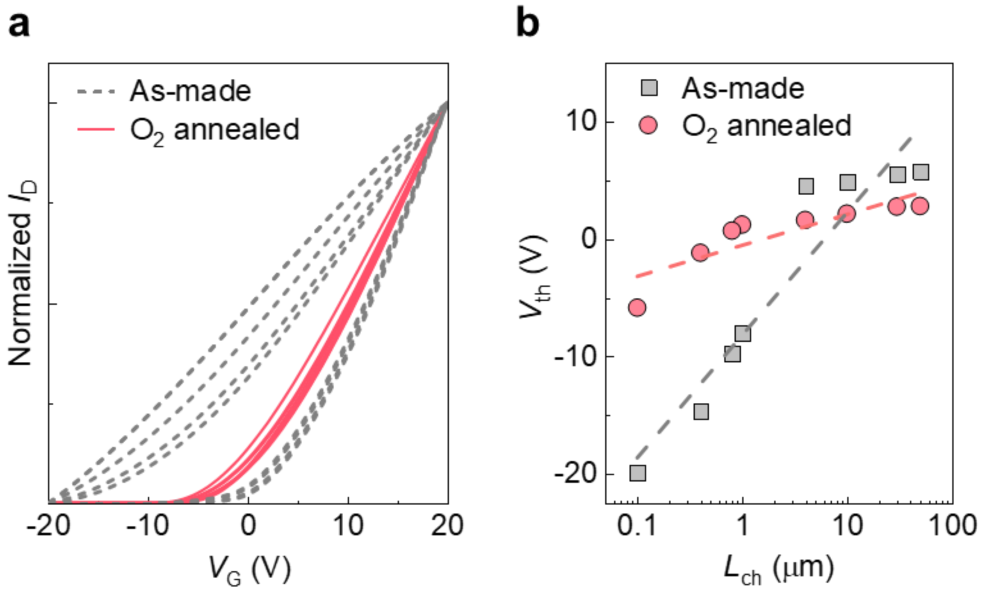

Figure S9. Postdeposition oxygen annealing mitigating the conductivity gradient in transistors. (a) Transfer characteristics of devices with different L_{ch} before and after O_2 annealing. (b) The V_{th} of devices with different L_{ch} before and after O_2 annealing. The $\partial V_{th}/\partial \log(L_{ch})$ changes from 10.4 to 2.6 after O_2 annealing.

Reference

(1) Kubota, W.; Utsunomiya, T.; Ichii, T.; Sugimura, H. Local current mapping of electrochemically-exfoliated graphene oxide by conductive AFM. *Jpn. J. Appl. Phys.* **2020**, *59*, SN1001.

(2) Tu, Y.; Utsunomiya, T.; Ichii, T.; Sugimura, H. Enhancing the electrical conductivity of vacuum-ultraviolet-reduced graphene oxide by multilayered stacking. *J. Vac. Sci. Technol. B* **2017**, *35* (3), 03D110.
